# Adjunctive intraocular and peri-ocular steroid (triamcinolone acetonide) versus standard treatment in eyes undergoing vitreoretinal surgery for open globe trauma (ASCOT): study protocol for a phase III, multi-centre, double-masked randomised controlled trial

**DOI:** 10.1186/s13063-016-1445-7

**Published:** 2016-07-22

**Authors:** Philip J. Banerjee, Victoria R. Cornelius, Rachel Phillips, Jessica W. Lo, Catey Bunce, Joanna Kelly, Caroline Murphy, Rhiannon Tudor Edwards, Elizabeth L. Robertson, David G. Charteris

**Affiliations:** Moorfields Eye Hospital NHS Foundation Trust, City Road, London, EC1V 2PD UK; National Institute for Health Research (NIHR) Clinical Research Facility at Moorfields Eye Hospital, London, UK; Imperial Clinical Trials Unit, School of Public Health, Imperial College London, London, UK; Department of Primary Care and Public Health Sciences, King’s College London, London, UK; National Institute for Health Research (NIHR) Biomedical Research Centre at Guy’s and St. Thomas’ NHS Foundation Trust and King’s College London, London, UK; NIHR Biomedical Research Centre at Moorfields Eye Hospital NHS Foundation Trust, London, UK; University College London Institute of Ophthalmology, London, UK; King’s Clinical Trials Unit, Institute of Psychiatry, Psychology and Neuroscience, King’s College London, London, UK; Bangor University, Bangor, UK

**Keywords:** Open globe trauma, Proliferative vitreoretinopathy, Visual acuity, ETDRS, Ophthalmology, Randomised controlled trial

## Abstract

**Background:**

Eyes sustaining open globe trauma are at high risk of severe visual impairment. Ocular injuries which result in visual loss invariably affect the posterior segment of the eye, and prevention of visual loss involves posterior segment (vitreoretinal) surgery. Despite improvements in vitreoretinal surgical techniques, outcomes in these patients remain unsatisfactory, and development of the intraocular scarring response proliferative vitreoretinopathy is the leading cause. Proliferative vitreoretinopathy is the most common cause of recurrent retinal detachment in these eyes; it is reported to occur in up to 45 % of cases.

**Methods/design:**

The Adjunctive Steroid Combination in Ocular Trauma (ASCOT) trial is a multi-centre, double-masked, parallel-arm randomised controlled trial with an internal pilot designed to investigate the effectiveness and cost-effectiveness of using intravitreal and sub-Tenon’s triamcinolone acetonide peri-operatively in patients undergoing vitrectomy following open globe trauma. In total, 300 eyes of 300 patients will be recruited and randomly allocated to one of two treatment groups. Both groups will receive standard surgical treatment and routine pre-operative and post-operative treatment and care. The treatment group will receive an adjunctive peri-operative steroid combination (triamcinolone acetonide) consisting of 4 mg/0.1 ml into the vitreous cavity and 40 mg/1 ml into the sub-Tenon’s space. The trial incorporates a two-stage internal pilot to examine projected recruitment and retention rates. Progression criteria from the internal pilot study will enable us to determine whether to undertake the main trial. Patients and primary outcome assessors will be masked to treatment allocation. The primary outcome will be an improvement from baseline to 6 months of at least 10 on the corrected visual acuity as measured by ETDRS letter score. Secondary outcomes will be development of scarring, retinal detachment, intraocular pressure abnormalities, quality of life and public sector service use.

**Discussion:**

This is the first powered, controlled clinical trial to investigate the use of adjunctive triamcinolone in patients undergoing vitrectomy following open globe trauma.

**Trial registration:**

EudraCT2014-002193-37. Registered on 5 September 2014.

ISRCTN30012492. Registered on 5 September 2014.

**Electronic supplementary material:**

The online version of this article (doi:10.1186/s13063-016-1445-7) contains supplementary material, which is available to authorized users.

## Background

Trauma is an important cause of visual impairment and blindness worldwide and a leading cause of blindness in young adult males [1]. Globally, it has been estimated that 1.6 million people are blind as a result of ocular trauma, with 2.3 million having bilateral low vision [2]. Ocular trauma is the most common cause of unilateral blindness in the world today, with up to 19 million individuals having unilateral blindness or low vision [2]. It is estimated that almost 1 million people in the United States live with trauma-related visual impairment [3]. Ocular trauma has extensive socio-economic costs: Patients with open globe injuries lose a mean of 70 days of work [4]. In the United States, work-related eye injuries costs over $300 million per year (http://www.preventblindness.org/), which equates to an annual cost to the U.K. economy (for which no comparable data exist) of £37.5 million.

In the United Kingdom, it is estimated that 5000 patients per year sustain eye injuries serious enough to require hospital admission, and that 250 of these will be permanently blind in the injured eye [5]. Recent European studies document incidences of 2.4 and 3.2 per 100,000 per year [6, 7] for open globe injuries, which suggests an annual incidence of between 1500 and 2000 for the United Kingdom.

Ocular injuries which result in visual loss frequently affect the posterior segment of the eye, and prevention of visual loss involves posterior segment (vitreoretinal) surgery. It is clear on the basis of recent published data that, although vitreoretinal surgical techniques have improved, outcomes remain unsatisfactory, and development of the intraocular scarring response proliferative vitreoretinopathy (PVR) is the leading cause of this [8–11].

### Proliferative vitreoretinopathy

Eyes sustaining penetrating or open globe trauma (OGT) are at high risk of severe visual impairment. Retinal detachment is common in these eyes, and multiple surgical interventions are often necessary. PVR is the most common cause of recurrent retinal detachment and visual loss in eyes with OGT. It is documented to occur in 10–45 % of all OGT cases [8–11], its incidence varying with the nature of the penetrating injury [8].

PVR is a process of fibrocellular scar tissue formation, which complicates 5–12 % of cases of primary retinal detachment, 16–41 % of cases of giant retinal tears and 10–45 % of cases of posterior segment trauma [12]. PVR represents a difficult vitreoretinal surgical challenge, and, although final retinal attachment may now be achieved, multiple surgeries are needed in many cases and visual results are frequently poor [12, 13]. Binocular vision outcomes are notably unsatisfactory in PVR [14]. PVR management is costly in terms of patient time and healthcare resources [13].

### Experimental data

Clinical observations and laboratory investigations undertaken on eyes with PVR and surgical specimens have identified potential targets for pharmacological adjuncts to surgical management of PVR [14]. The cellular components of PVR peri-retinal membranes (retinal pigment epithelial, glial, inflammatory and fibroblastic cells) proliferate and may also be contractile, and they are thus targets for anti-proliferative agents. There is a notable inflammatory component to the PVR process, with marked blood-retinal barrier breakdown and a greater tendency to intraocular fibrin formation [15]. Macrophages and T lymphocytes have been identified in PVR membranes [14], and, although relatively small in number, they may play an important role in membrane development and contraction through growth factor production. Thus, both cellular proliferation and the intraocular inflammatory response are realistic targets for adjunctive treatments in PVR.

Corticosteroid treatment can potentially influence both the inflammatory and proliferative components of PVR. Experimental work has suggested that the corticosteroid triamcinolone acetonide can reduce the severity of PVR [16]. In addition, it has been demonstrated that peri-ocular corticosteroids can reduce the severity of experimental PVR [17]. Laboratory work has also demonstrated that triamcinolone appears to have no significant retinal toxicity [18], although in vitro it downregulates the proliferation of retinal cells.

### Clinical data

Clinically, intravitreal triamcinolone acetonide (IVTA) has recently been extensively used to treat macular oedema, intraocular inflammation and sub-retinal neovascularisation without demonstrable retinal toxicity, but with a notable incidence of raised intraocular pressure (IOP) and cataracts. Previous small-scale, uncontrolled clinical studies of PVR have suggested that systemic prednisolone [19], infused dexamethasone [20] and IVTA [21–23] may reduce the severity of PVR. An external, single-centre pilot randomised controlled trial (RCT) investigating intravitreal and sub-Tenon’s triamcinolone as an adjunctive treatment in eyes undergoing pars plana vitrectomy (PPV) following OGT [24] suggested an improved visual outcome in eyes receiving adjunctive corticosteroid treatment and supported an adequately powered RCT to provide a definitive answer [24].

### Investigational medicinal product triamcinolone acetonide

Triamcinolone is a hydrophobic, long-acting corticosteroid which is licensed for intra-articular use to treat arthritis and for intramuscular use in a variety of systemic inflammatory conditions. Ophthalmologists have experience using triamcinolone off-license via peri-ocular administration for over 50 years, with administration via the intraocular route adopted for over 30 years. It has been used to treat a variety of posterior segment ocular inflammatory pathologies [25–28]. Its use to visualise the posterior hyaloid during PPV has been well established [29]. Additionally, IVTA has been found to reduce post-operative inflammation following vitrectomy surgery [30]. It has been investigated specifically to determine its effect on vitreoretinal scarring, with varying success [21–23]. It has an extremely well-documented safety profile, with the most common side effect recorded as elevated IOP [31]. Data derived from a recently published external pilot study [24] showed a similar incidence of elevated IOP between both groups: 35 % (*n* = 7) of patients who received IVTA compared with 25 % (*n* = 5) of those patients who received standard care.

### Objectives

#### Internal pilot objectives

1. To ascertain recruitment rates and retention in the first 6 months of the study being open to recruitment
2. To verify the number of eligible participants across centres
3. To assess the implementation of the trial and adherence to the study protocol procedures in non-teaching and teaching hospitals
4. To satisfy the criteria for proceeding to the main trial

#### Primary objective

The aim of the main study is to determine whether adjunctive intraocular and peri-ocular steroid (triamcinolone acetonide) treatment improves visual acuity (VA) at 6 months compared with standard treatment in eyes undergoing vitreoretinal surgery for OGT.

#### Secondary objectives

Secondary objectives of the study are to determine whether adjunctive intraocular and peri-ocular steroid (triamcinolone acetonide) treatment improves the development of scarring (PVR), retinal detachment, IOP abnormalities and other complications in eyes undergoing surgery for OGT. In addition, we will assess the effects of treatment on quality of life as measured with the EQ-5D and 25-item Visual Function Questionnaire tools, as well as public sector service use to undertake an economic evaluation using the Client Service Receipt Inventory.

## Methods/design

### Study design

The Adjunctive Steroid Combination in Ocular Trauma (ASCOT) study is a multi-centre, parallel-arm RCT in which patient and outcome assessor are masked. The trial will test the superiority of the intervention at 6 months after vitrectomy surgery. A structured, internal, two-stage pilot with clear stop and go criteria is included. In the pilot, we will examine projected recruitment and retention rates. Pre-specified progression criteria for each stage of the internal pilot will enable us to decide whether to undertake the main trial, and data from this two-stage internal pilot will contribute to the final analysis.

### Study population

Patients with an open globe injury undergoing vitrectomy as either a primary or secondary procedure are the study population being investigated. OGT is classified as one of the following: (1) a full-thickness eyewall injury in the form of a rupture caused by a blunt object or (2) a laceration caused by a sharp object or an intraocular foreign body. As the study intervention remains investigational, in patients with bilateral eye injuries, the eye with the worse injury (i.e., the eye with the poorest visual potential according to the clinician’s discretion) will be considered as the study eye for randomisation, and the better eye will receive standard treatment. We expect this to be a rare occurrence and are therefore not stratifying by binocularity.

Patients will be eligible for inclusion if they meet the following criteria:

1. Adult patients (aged 18 years or over at the time of enrolment)
2. Full-thickness, OGT undergoing vitrectomy
3. Ability to give written informed consent
4. Willingness to accept randomisation and attend follow-up for 6 months

Patients will be excluded if they meet the following criteria:

1. Pre-existing uncontrolled uveitis
2. Definitive diagnosis of previous steroid-induced glaucoma
3. Pregnant or breastfeeding females
4. Allergy or previous known adverse reaction to triamcinolone acetonide
5. Current or planned systemic corticosteroid use of a dose above physiological levels (e.g., >10 mg prednisolone)

### Informed consent

Informed consent will be obtained by a suitably qualified and experienced individual who has been delegated this duty by the chief investigator (CI) or the principal investigator (PI) (site-specific) on the delegation log. Rarely, eligible patients may present for emergency surgery out of hours or on occasions when the PI or a delegated individual is not on-site. In such cases, informed consent may be taken by individuals who are aware of good clinical practice (GCP) and familiar with key aspects of the study.

Informed consent will be obtained before any trial-specific procedures are completed (i.e., those that are outside routine clinical care). Clinical findings documented during an ocular assessment that has been performed as part of routine clinical care may be used to populate the baseline Case Report Form (CRF), provided the assessment is performed within 14 days before the study intervention.

### Randomisation

The randomisation procedure is performed intraoperatively at the time of the study vitrectomy, after final confirmation that the retina is attached. Randomisation is conducted via a telephone service to the Emergency Scientific and Medical Services (ESMS) global service hosted at the King’s Clinical Trials Unit (KCTU) at King’s College London. All randomised patients must first be registered in the study electronic Case Report Form (eCRF) system to be generated a unique patient identification number (PIN). This will be undertaken by the study team prior to the patient’s going to the surgical theatre, but in some cases the ESMS Global service may also assign a study PIN if no member of the research team is available. Patients will be randomised in a 1:1 allocation ratio at the level of the individual using random permuted blocks, with stratification by trial site.

### Interventions

Patients will be randomised to the treatment group or the control group:

1. *Treatment group*: 4 mg/0.1 ml of IVTA will be injected into the vitreous cavity following closure of the scleral ports at the end of procedure, and 40 mg/1 ml of triamcinolone acetonide will be given as a sub-Tenon’s injection at the end of the procedure.
2. *Control group*: No additional adjunctive corticosteroid medication will be administered, and standard care will be given.

All patients will be advised to continue their routine concomitant medications throughout the study, as there is an extremely low likelihood of the investigational medicinal product (IMP) reaching systemic concentrations high enough to cause interactions.

The following non-investigational medicinal products (NIMPs) may be used by the patients in both groups in their operated eye as part of their normal routine care:

1. *Pre-operatively*: Pupil-dilating drops may be instilled. Local policy on pre-operative dilating agents will be followed, but examples may include but are not restricted to gutte cyclopentolate and gutte phenylephrine.
2. *Peri-operatively*: Sub-conjunctival antibiotics will be administered at the surgeon’s discretion in line with the local pharmacy formulary (e.g., cefuroxime [Zinacef; GlaxoSmithKline, Uxbridge, UK] 125 mg or gentamicin in β-lactam-sensitive individuals). Sub-conjunctival steroid injections may be administered at the surgeon’s discretion (e.g., 4 mg dexamethasone or 4 mg betamethasone).
3. *Post-operatively*: Routine post-operative topical antibiotics and cycloplegic drops may be used at the discretion of the operating surgeon (e.g., gutte chloramphenicol 0.5 % four times daily for 2 weeks, gutte cyclopentolate 1.0 % three times daily for 1 week). Routine post-operative topical steroids may be used at the surgeon’s discretion (e.g., gutte dexamethasone 0.1 % or gutte prednisolone acetate 1 % with duration and frequency depending on the level of post-operative inflammation).

The above NIMPs are detailed as a guide only. Local policy for pre-, peri- and post-operative medications will be followed and remain at the discretion of the operating surgeon.

### Outcomes

#### Internal pilot progression criteria

##### Stage 1: Funded study months 1–12 (recruitment month 6)

It is planned that five study sites will be set up by month 4 and ten study sites by month 5. It is anticipated that they should be recruiting participants to the internal pilot at the start of month 6. The trial is to proceed to stage 2 of the internal pilot if at funded study month 12 the following are true:

1. Ten study sites are set up by month 10
  *and*
2. At least 30 participants have been recruited to the trial during the first 6 months of the recruitment period

##### Stage 2: Funded study months 12–18 (recruitment month 12)

It is planned that 20 study sites will be set up by study month 12 and are able to recruit participants at the start of month 13. The trial is to proceed to full trial if at funded study month 18 the following are true:

1. Less than 7 of 30 or less than 8 of 40 stage 1 internal pilot participants have withdrawn from the trial by their 6-month follow-up appointment
  *and*
2. An additional 48 participants have been recruited to the trial during recruitment months 6–12

If the progression criteria for either stage 1 or stage 2 are not met due to an inadequate number of study sites being open, the reasons for these delays will be examined and discussed with the funders. Reasons for failure to meet recruitment and retention rate will be examined in detail, and the feasibility of the trial will be assessed in light of the information obtained from the internal pilot. If appropriate, the recruitment targets will be redrawn, and further study sites will be added to the trial in order to meet sample size requirements within the funding period.

#### Main study outcomes

The primary outcome is the proportion of patients with a clinically meaningful improvement of at least 10 on the corrected VA in the study eye measured using validated Early Treatment Diabetic Retinopathy Study (ETDRS) vision charts at a starting distance of 4 m from baseline to 6 months after initial surgery.

The following are secondary outcomes of the study:

1. Total ETDRS score in the study eye at the 6-month follow-up appointment
2. The proportion of patients in whom retinal detachment with PVR occurs at any time point within 6 months of the study vitrectomy^1^
3. The proportion of patients in whom stable complete retinal reattachment (without internal tamponade present) is achieved at 6 months post-study vitrectomy^1^
4. The proportion of patients in whom stable macular retinal reattachment (without internal tamponade present) is achieved at 6 months post-study vitrectomy^1^
5. The proportion of patients in whom a tractional retinal detachment occurs at any time point within 6 months of the study vitrectomy^1^
6. The number of operations to achieve stable retinal reattachment (either complete or macula) at 6 months after the study vitrectomy^1^
7. The proportion of patients with hypotony (<6 mmHg) at any time point within 6 months of the study vitrectomy
8. The proportion of patients with raised IOP (>25 mmHg) at any time point within 6 months of the study vitrectomy
9. The proportion of patients who develop macular pucker by 3 and 6 months and/or require macular pucker surgery at any time point within 6 months of the study vitrectomy
  Items 1–9 above apply to the study eye only.
10. Use of public sector resources:
  - *Client Service Receipt Inventory (CSRI)*: Primary and secondary health and social care service use will be recorded using a brief CSRI created for the study. At baseline, patients will be asked to recall service use in the last 4 weeks, and at 3 and 6 months patients will be asked to recall their service use for the previous 3 months.
11. Quality of life (QoL) as measured with the following instruments:
  - *EQ-5D-5L* [32]: The EQ-5D is a generic, preference-based, health-related quality of life measure.
  - *Visual Function Questionnaire (VFQ-25)* [33]: The VFQ-25 measures vision-related QoL.

### Sample size and recruitment

Published data [34] indicate that the distribution of the best corrected VA ETDRS letter score at 6 months will be skewed, which is in line with results from the external pilot RCT [24]. In the external pilot trial, the majority of patients (35 of 40) had a score of 0 for VA baseline values, and the shape of the distribution of VA at 6 months was non-identical between the treatment group and the control group. Both of these factors impact the choice of suitable methods for analysis and thus an appropriate approach to calculating the sample size.

As a result of a small mean difference and non-identical distributions observed in VA between treatment groups in the external pilot study, we chose to define the primary outcome to be a clinically meaningful improvement in VA of 10 letters or more in the treatment group. A change of 10 letters is widely accepted to be clinically meaningful in research studies of eye disease [13, 15, 19–22, 35–38].

With 140 patients per group and a significance value of 5 %, we would have 90 % power to detect a 19 % increase (from 55 % to 74 %) in patients who have a meaningful minimum improvement in VA of at least 10. The expected proportion in the control group is based on the external pilot data, where we observed 55 % of patients gaining 10 ETDRS letters after vitrectomy surgery for OGT [24]. Equivalently, with 140 per patients per group and using a 5 % significance level, we would have 85 % power to detect a 17 % increase (from 55 % to 72 %) in patients who have a meaningful minimum improvement in VA of at least 10.

Previous trials run by Moorfields Eye Hospital and involving tertiary teaching hospitals had no more than a 5 % dropout rate at 6-month follow-up [24, 35, 37, 38]. As our multi-centre trial includes non-specialist centres, we anticipate the dropout rate will be higher. Therefore, allowing for a 7 % dropout rate, we aim to recruit 300 patients to participate in the trial. All 300 patients will be identified and recruited from outpatient clinics and emergency referrals at one of the specified U.K. study sites.

### Masking

Patients and primary outcome assessors will be masked to treatment allocation. Outcome assessors may consist of technicians, nursing staff or healthcare assistants who are familiar with measuring VA using the ETDRS chart. The operating surgeon is masked until the end of the study operation at the point of randomisation.

### Emergency unmasking

Within the first few days after IMP administration, there is a possibility that the intraocular triamcinolone may migrate into the anterior chamber. This may appear as a pseudohypopyon, and the clinical picture could mimic endophthalmitis. It is expected that patients will be followed at their treatment site. Operating surgeons will be encouraged to anticipate this occurrence and communicate this to the reviewing team in the immediate post-operative period. However, it is possible (although unlikely) that patients may attend a unit other than their study site within the early post-operative period. In this circumstance, a treating clinician should be made aware of the patient’s treatment allocation if the patient suspects a diagnosis of endophthalmitis, such that (1) the patient is not subjected to unnecessary invasive interventions where the findings are of an innocuous pseudohypopyon or (2) urgent treatment is withheld under the false premise that the observed hypopyon is a pseudohypopyon.

The study code should be broken only for valid medical or safety reasons highlighted in the circumstances above where it is necessary for the investigator or treating healthcare professional to know which treatment the patient is receiving before the patient can be treated as per standard clinical care. Subject always to clinical need, where possible, members of the study site research team should remain masked. The procedures to be used for unmasking during and out of office hours by study physicians and other treating physicians follows a standard operating procedure (SOP) and the code breaks for the trial are held by the ESMS global service.

### Data collection

#### Screening

Patients who are invited to attend the hospital specifically for the purposes of screening will be asked to sign a written consent form by a member of the study team or a delegated individual. A full medical and ophthalmic history will be obtained to confirm eligibility.

#### Baseline assessments

Baseline assessments will be performed within 14 days prior to the study vitrectomy. Data collected as part of routine clinical care may be used to populate the baseline CRF prior to informed consent, but the patient will not be registered on the eCRF, and no data will be entered into the eCRF system until the patient has signed a consent form. The eCRF system will generate a unique study identifier for all patients consented and screened for participation in the study. The method of data collection will be a combination of medical history, applanation tonometry, slit-lamp biomicroscopy or indirect ophthalmoscopy, and intraoperative findings. QoL data will be collected using the ED-5Q and VFQ-25 tools and a CSRI questionnaire. Screening and baseline assessments may occur concurrently.

#### Subsequent assessments

Patient follow-up will mirror the schedule of standard National Health Service (NHS) care with assessments at 3 months and 6 months post-surgery. Table 1 is a guide for an expected schedule of visits. Data entry time points will be at (1) baseline, (2) study vitrectomy, (3) month 3 and (4) month 6, with a 4-week time window allowed on either side of the scheduled visit (refer to shaded columns in schedule of visits in Table 1).

**Table 1:**
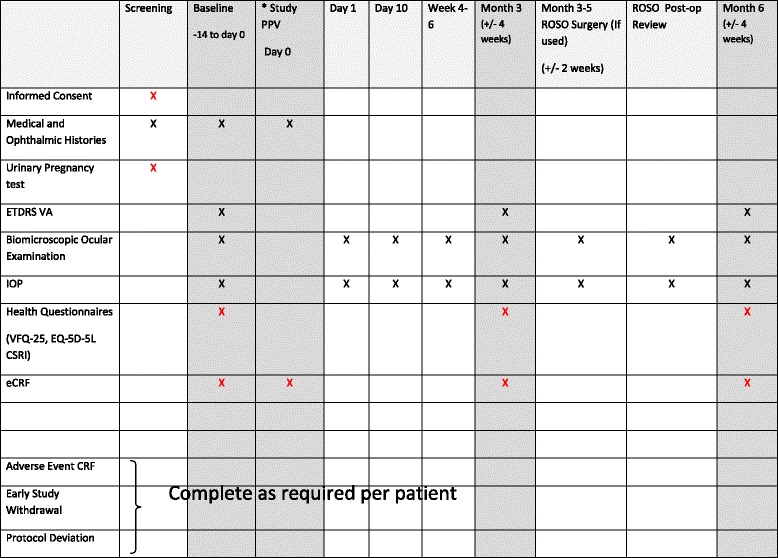
Study assessments

The table may be used as a guide and represents routine standard care. Patients may require fewer visits (e.g., if silicone oil tamponade is not used, or more frequent visits as their clinical need arises, such as reoperations). The restrictions on time windows allowed for scheduled visits relate only to data entry points and are highlighted as shaded columns in the table (i.e., baseline, months 3 and 6)

Shaded columns = data collection points (i.e., eCRF completion required); black X = performed as part of routine NHS care; red X = performed in addition to routine care as part of study

*Abbreviations*: *ETDRS VA* Early Treatment Diabetic Retinopathy Study visual acuity, *IOP* intraocular pressure, *VFQ-25* 25-itemVisual Functioning Questionnaire, *EQ-5D* EuroQol health questionnaire, *CSRI* Client Service Receipt Inventory, *CRF* case report form, *PPV* pars plana vitrectomy, *ROSO* removal of silicone oil

^a^Investigational medicinal product administration in treatment group

#### Discontinuation/withdrawal of participants

Participants will be withdrawn from the study in the following circumstances:

1. The administration of an intraocular or peri-ocular corticosteroid is deemed necessary by the patient’s consultant. (The use of intraocular triamcinolone as a surgical adjunct to visualise the hyaloid is permitted in both groups, as it is subsequently cleared intraoperatively. The likelihood of therapeutic doses remaining at the end of the procedure is considered to be extremely low.)
2. The patient is commenced on systemic corticosteroids at a dose higher than physiological levels (e.g., >10 mg oral prednisolone).
3. The patient expresses a wish to withdraw from the trial.

Withdrawn subjects will not be replaced, as attrition has been accounted for in the sample size calculation. Participants who wish to withdraw will be asked if they are willing to undertake VA assessment at 6 months and supply other study data collected at this routine visit.

### Data management

Study data will initially be recorded on a source data worksheet and then transcribed to the eCRF system (InferMed MACRO; Elsevier, London, UK) hosted at the KCTU at King’s College London. It will be the responsibility of the investigator to ensure the accuracy of all data entered in the eCRFs. InferMed MACRO eCRF version 4 will be used to record the study data.

All data will be handled in accordance with the U.K. Data Protection Act 1998. The eCRFs will not bear the patient’s name or other personally identifiable data. The subject’s initials, date of birth and patient identification number will be used for identification. Source data worksheets will be completed for each patient but will not be removed from the recruiting study sites. Signed consent forms will be filed in the investigator site file.

A trial-specific monitoring plan will be established for the study as part of the oversight planning. The trial will be monitored with the agreed plan. The trial manager will raise data discrepancies within the system, and sites will respond to each before the discrepancy is closed. At the end of the trial, once all queries are resolved and all data fields are completed with data or missing data codes (where applicable), the database will be locked for analysis, and this process will be overseen by the KCTU.

### Statistical methods

#### Summary of baseline data and flow of patients

A Consolidated Standards of Reporting Trials (CONSORT) flow diagram will be produced to show the number of eligible patients, the number recruited and randomised, and the number withdrawing with reasons for withdrawal [39]. Baseline characteristics will be tabulated and summarised by treatment arm. The primary analysis will follow an intention-to-treat principle as specified in the International Conference on Harmonisation (ICH) E9 guideline [40], whereby all participants are analysed in the arm to which they were allocated, regardless of subsequent procedures. The proportion of patients with an improvement in VA score of 10 or more in the study eye, as measured with the ETDRS vision chart, will be compared between treatment groups.

#### Primary outcome analysis

The proportion of participants with improvement in VA of 10 letters of more (yes or no) will be tabulated by treatment arm and time point. Initially, we will calculate an unadjusted difference in proportion at 6 months between treatment arms with 95 % confidence intervals. We will obtain the adjusted treatment effect estimate by fitting a generalised linear model with improvement in VA of 10 letters of more (yes or no) as the outcome and treatment group and baseline ETDRS value as covariates. The treatment effect estimate will be reported with a two-sided 95 % confidence interval and corresponding *p* value. We will use a generalised linear model with binomial distribution and logit link function to calculate the OR with associated 95 % confidence intervals. If many centres recruit a small number of participants (e.g., *n* < 5), a sensitivity analysis exploring suitable methods to adjust for centre in the model will be undertaken [41].

#### Secondary outcome analysis

We will undertake analysis of the secondary outcomes in a similar manner to the primary analysis described above. Secondary outcomes will be summarise and tabulated by treatment arm and time point. We will estimate and test for a difference between treatment arms for each endpoint specified in the secondary outcomes listed above. Initially, we will calculate an unadjusted difference and 95 % confidence interval. A suitable generalised linear model will then be fitted for each outcome The logit link and binomial distribution will be used for binary outcomes, the identity link and Gaussian distribution will be used for continuous outcomes, and the log link and Poisson distribution will be used for count outcomes, which will include an overdispersion parameter if required. Similarly to the primary outcome analysis, the models will include treatment group, baseline ETDRS value and centre as covariates where appropriate. We will assess all outcomes at the 6-month time point.

Adverse events (AE), adverse reactions (AR), serious adverse events (SAE) and serious adverse reactions (SAR) will be summarised. AE will be tabulated by treatment arm for both the number of events and the number of participants with events. No formal comparisons will be made.

All statistical tests and confidence intervals will be two-sided. Statistical significance will be considered at the 5 % level, and confidence intervals will be at the 95 % level [42].

#### Health economics analysis

We will analyse the incremental cost-effectiveness of the trial intervention (intraocular and peri-ocular steroid) in eyes undergoing vitreoretinal surgery for ocular trauma compared with surgery alone in terms of changes in VA. To enable this analysis, we will, from a public sector, multi-agency perspective [42–44]:

1. Fully cost the vitreoretinal surgery and follow-up
2. Record patients’ primary and secondary care health service use and social care use over the 6-month follow-up (using a research nurse interviewer-administered CSRI, costed using national unit costs [45, 46], and making use of routine hospital data on surgical and post-operative care as part of the CRF)
3. Conduct a primary cost-effectiveness analysis (using the trial primary outcome measure of VA as our measure of effectiveness)
4. Conduct a secondary cost-consequence analysis (to take account of impact on wider effects such as employment and VA), including calculation of quality-adjusted life-years (QALYs) using the EQ-5D questionnaire as our measure of utility to generate a cost per QALY for comparison with the National Institute for Health and Care Excellence ceiling of £20,000–£30,000 [47]
5. Through bootstrapping, generate cost-effectiveness acceptability curves to communicate to policy makers the probability that the intervention is cost-effective [48]
6. Undertake sensitivity analysis to explore uncertainty by varying key assumptions in our analysis

A detailed statistical/health economics analysis plan has been developed and approved by the Trial Steering Committee (TSC). Masked statisticians will be responsible for overseeing all the statistical aspects of the trial, and a sub-group unmasked statistician will be responsible for undertaking interim analysis for Data Monitoring and Ethics Committee (DMEC) reports and final study analysis.

### Data monitoring

The overall management structure of this study will consist of the Trial Management Group (TMG), the TSC and a DMEC. The TMG will be responsible for the day-to-day running and management of the trial. The TSC will ensure the overall integrity of the study by monitoring its progress and taking account of regular reports from the DMEC and TMG. The TSC will consist of an independent chair and other members including an independent retinal specialist, a trauma specialist and a patient and public involvement representative. The TSC is expected to meet annually (or more often, if determined by the chair). The DMEC will independently monitor the trial data to ensure that the trial is being implemented in accordance with the highest standards of patient safety and ethical conduct according to a pre-planned charter based on the DAMOCLES Study Group report [49, 50]. Throughout the trial, the DMEC will monitor data on recruitment, AE, emerging external evidence, sample characteristics and primary outcome, and will make recommendations.

### Adverse events and safety reporting

Safety reporting will adhere to the sponsor’s SOPs, and monitoring, recording and reporting of AE will be carried out in line with the Medicines and Healthcare products Regulatory Agency (MHRA) guidelines. AE will be recorded with clinical symptoms and accompanied with a simple, brief description of the event, including dates as appropriate. AE will be reportable to the sponsor for each patient for the duration of the patient’s participation in the trial as per the protocol.

#### Ocular AE

All ocular AE reported by patients and observed by the study team will be recorded in the medical records and the eCRF following randomisation until the patient has completed the final study visit at 6 months, with the exception of some events which are inevitable consequences of the surgical intervention and extremely unlikely to be IMP-related. These events will only be recorded in the medical records and will be discussed further herein.

The following expected ocular events will be considered AE and will be actively monitored for by the investigators: elevated IOP, hypotony (IOP <6 mmHg), pseudohypopyon, retinal detachment, further ocular surgery, endophthalmitis, scleritis, uveitis and rubeosis. AE will be recorded regardless of whether they are considered to be drug-related or expected or unexpected. The recording of severity of raised IOP will be done as follows:

1. *Mild*: >25 mmHg but <35 mmHg
2. *Moderate*: ≥35 mmHg
3. *Severe*: Any interventional invasive procedure (e.g., surgery/laser) required to control the elevated IOP acutely or long-term during the study period

Examples of ocular events that will not be considered AE on the basis of being expected findings in this disease population, and which as such will only be recorded in the medical notes, include sub-conjunctival haemorrhage, conjunctival chemosis, peri-orbital oedema, routine post-operative pain, cataract, and corneal epithelial defect secondary to intraoperative epithelial debridement. If any of the above events occur with a severity or duration that is unexpected by the PI, they may then be reported as AE with clear reasons given (e.g., ‘prolonged post-operative pain’ or ‘severe sub-conjunctival haemorrhage’). All ocular events that meet the definition of SAE will be recorded in the medical notes, the eCRF and the SAE log.

#### Non-ocular AE

As the IMP is administered locally and the systemic absorption negligible, the likelihood of a non-ocular event being related to IMP administration is extremely low [36]. As this trial is an effectiveness trial, detailed AE reporting on non-ocular events is not required to answer the research question. Therefore, if a non-ocular AE occurs, it will be recorded in the medical notes and logged on the eCRF only if it may be considered an AR (i.e., possibly, probably or definitely IMP-related). All non-ocular events that meet the definition of SAE will be recorded in the medical notes but will be recorded in the eCRF and SAE log only if they are possibly, probably or definitely IMP-related (i.e., a SAR).

All SAEs are immediately reportable to the sponsor (within 24 h of the investigator becoming aware of it). All suspected unexpected serious adverse reactions (SUSARs) will be processed by the sponsor, which will notify the research ethics committee (REC) and MHRA within 7 calendar days if fatal or life-threatening or 15 calendar days otherwise. The sponsor will prepare a report on the SUSAR with the CI and submit the completed report to the SUSAR system. The sponsor, together with the CI and trial manager, will inform the PIs, DMEC and REC with a copy of the report. The assessment of relationship of AEs to the administration of IMP is a clinical decision made by the investigators on the basis of all available information at the time of the completion of the case report form.

The study will be conducted in accordance with the ICH GCP guideline [51], as set out in the European Union Clinical Trials Directive [52] and associated U.K. regulations [53] and all subsequent amendments. The study will comply at all times with the Declaration of Helsinki [54]. This protocol is reported in accordance with the Standard Protocol Items: Recommendations for Interventional Trials (SPIRIT 2013) checklist (see Additional file 1) [55].

### Declaration of interests

All those listed as authors are qualified for authorship, and all who are qualified to be authors are listed as authors on the byline. No conflicts of interest, financial or other, exist.

### Publication policy

The results of this study will be submitted for publication in peer-reviewed medical journals, regardless of whether the findings are in favour of the trial intervention.

## Discussion

This aim of this multi-centre RCT investigating the use of a triamcinolone acetonide is to test the hypothesis that adjunctive triamcinolone acetonide, given at the time of surgery, can improve the outcome of vitreoretinal surgery for OGT. We will specifically analyse the influence of the study intervention on VA, the incidence of retinal detachment and the development of scarring (proliferative vitreoretinopathy) in patients with OGT. Side effects and complications will be monitored and reported. We will undertake QoL assessments of the study patients, and a cost-effectiveness analysis will be carried out.

We have based the projected recruitment on data derived from the external pilot study at the principal study site, in addition to projections provided by participating sites following local internal audits. Due to the poor prognosis associated with current standard treatment, we expect a high recruitment uptake following successful eligibility screening as observed in the external pilot. However, we have designed the study with a two-stage internal pilot to assess recruitment and retention. Progression criteria for each stage of the internal pilot will determine the decision whether to undertake the main trial, and data from this two-stage internal pilot will contribute to the final analysis.

We accept that, as our eligibility criteria are inclusive and relatively unrestricted, it is likely that the cohort will be a heterogeneous group. The severity of open globe injury may range from a relatively minor injury at one end of the spectrum (i.e., a small anterior corneal wound and secondary lens capsule breach requiring a posterior vitreolensectomy) to a severe posterior globe rupture with total retinal detachment and extensive suprachoroidal haemorrhage. However, we expect the adequacy of randomisation to compensate for this and shall acknowledge any unequal weighting within the groups as limitations of the study. However, we accept that we may limit our sensitivity analysis to small differences between the two groups.

The authors have designed the study to reduce investigator bias by masking the primary outcome assessors, the patients and the operating surgeons (until the point of randomisation). The investigators are not formally masked to the treatment allocations, as the IMP is sometimes visible on posterior chamber assessment for up to 4 weeks. However, data entry points for secondary outcomes are at 3 and 6 months post-injection, and thus knowledge of individual patients’ treatment allocation at these time points is unlikely.

In summary, this is the first powered RCT to investigate the use of triamcinolone in patients undergoing vitrectomy surgery following OGT, with an accompanying economic evaluation.

## Trial status

The authors confirm that the trial remained in active recruitment as of the 14th July 2016.

## Abbreviations

AE, adverse event; AR, adverse reaction; CI, chief investigator; CONSORT, Consolidated Standards of Reporting Trials; CRF, Case Report Form; CSRI, Client Service Receipt Inventory; DMEC, Data Monitoring and Ethics Committee; eCRF, electronic Case Report Form; ESMS, Emergency Scientific and Medical Services; ETDRS, Early Treatment Diabetic Retinopathy Study; GCP, good clinical practice; IMP, investigational medicinal product; IOP, intraocular pressure; IVTA, intravitreal triamcinolone acetonide; KCTU, King’s Clinical Trials Unit; MHRA, Medicines and Healthcare products Regulatory Agency; NIMP, non-investigational medicinal product; OGT, open globe trauma; PI, principal investigator; PIN, patient identification number; PPV, pars plana vitrectomy; PVR, proliferative vitreoretinopathy; QALY, quality-adjusted life-year; QoL, quality of life; RCT, randomised controlled trial; REC, research ethics committee; SAR, serious adverse reaction; SAE, Serious adverse event; SOP, standard operating procedure; SPIRIT, Standard Protocol Items: Recommendations for Interventional Trials; SUSAR, suspected unexpected serious adverse reaction; TMG, Trial Management Group; TSC, Trial Steering Committee; VA, visual acuity; VFQ-25, 25-item Visual Function Questionnaire

## Additional file

Additional file 1: SPIRIT 2013 Checklist: recommended items to address in a clinical trial protocol and related documents. (DOC 114 kb)

## Footnotes

1 Assessed by clinician examining the patient as part of usual clinical visits.
